# Prefrontal cortex activation patterns in age-related cognitive decline: insights from verbal fluency task

**DOI:** 10.3389/fneur.2026.1691856

**Published:** 2026-01-23

**Authors:** Qiaoxia Zhen, Ruifeng Sun, Conglin Han, Jilin Wu, Li Song, Weijun Gong

**Affiliations:** 1 Department of Neurological Rehabilitation, Beijing Rehabilitation Hospital, Capital Medical University, Beijing, China; 2 Capital Medical University, Beijing, China

**Keywords:** age-related cognitive decline, executive function, functional near-infrared spectroscopy, prefrontal cortex, verbal fluency task

## Abstract

**Objective:**

Age-related cognitive decline (ARCD) is highly prevalent in aging populations and is characterized by progressive declines in cognitive function—particularly executive function (EF), which merits focused investigation. This study aimed to compare prefrontal cortex (PFC) activation patterns between ARCD patients with executive dysfunction (ED) and those with non-executive dysfunction (non-ED) using functional near-infrared spectroscopy (fNIRS) during a verbal fluency task (VFT). It further explored correlations between alterations in PFC activation, the severity of EF impairment, and global cognitive function.

**Methods:**

A total of 36 elderly individuals diagnosed with ARCD were recruited for this study. Participants were stratified into the ED group or the non-ED group based on neuropsychological test performance, with 18 individuals in each group. fNIRS was employed during the VFT to assess cortical activity. Additionally, a comprehensive neuropsychological assessment was conducted to evaluate global cognitive function, as well as specific domains related to memory and EF. Correlations between PFC activation, as reflected by changes in oxyhemoglobin (oxy-Hb) concentration and cognitive outcomes were analyzed.

**Results:**

The participants of the ED group were older than those in the non-ED group, and had a higher incidence of type 2 diabetes mellitus than those in the non-ED group. The fNIRS-VFT analysis revealed no activation of the PFC in the ED group (*p >* 0.05), whereas only minimal activation of the left frontal pole was observed in the non-ED group of ARCD (FDR-corrected *p <* 0.05). Specifically, activation at Channel 13 (located at the left frontal pole) was statistically higher in the non-ED group than in the ED group at the uncorrected level (*p* < 0.05); however, this difference became non-significant after FDR correction (*p* > 0.05). Additionally, the Montreal Cognitive Assessment (MoCA) score was significantly correlated with oxy-Hb concentration changes at Channel 21 (located at the left frontal pole, *r_s_* = 0.515, FDR-corrected *p* < 0.05).

**Conclusion:**

ARCD showed attenuated PFC activation during VFT, with no significant difference between the ED and non-ED groups. The decline in PFC activation was correlated with lower MoCA scores, suggesting that fNIRS-derived PFC activation metrics might serve as a potential biomarker for global cognitive decline in ARCD.

## Introduction

With the accelerating aging process in China, the incidence of ARCD has been on the rise, becoming an increasingly prominent challenge (1). Specifically, the incidence has reached 20% (2). ARCD refers to a syndrome characterized by a measurable decline in cognitive performance closely associated with physiological brain aging—defined operationally as a 1 – standard deviation (SD) reduction below the normative mean of cognitive assessment domains—yet crucially, it does not impair an individual’s activities of daily living. Distinct from mild cognitive impairment (MCI) (typically defined as a ≥1.5 SD decline below normative means), ARCD is demarcated from MCI by one core criterion: the absence of documented evidence of underlying cerebral or systemic diseases/conditions known to induce cerebral dysfunction (3, 4). Normal aging is associated with neuroanatomical and physiological changes that can impair specific cognitive domains, particularly working memory, processing speed, and EF (5, 6).

Executive function, known as cognitive control, includes flexibility, planning, working memory, task-switching, and inhibitory control (7, 8). Unlike fundamental cognitive domains—such as working memory and processing speed, which function as discrete, bottom-up information-processing modules—EF serves as a central integrative regulator that orchestrates and deploys these basic cognitive abilities to execute sophisticated, goal-directed behaviors. Neuroanatomically anchored in the PFC and its distributed cortical-subcortical networks (7), EF is not merely a constituent of the broader cognitive system but rather the linchpin that translates raw cognitive capacity into real-world functional competence. Critically, across all cognitive domains, EF has been identified as the sole and strongest predictor of functional independence, as operationalized by Instrumental Activities of Daily Living (IADL)—a key proxy for independent living encompassing complex instrumental tasks (9). In elderly individuals with ARCD, this unique, domain-specific association underscores that even minimal EF impairments can compromise IADL performance, even in the presence of preserved fundamental cognitive processes. Therefore, EF warrants attention in individuals with ARCD. Current EF assessment relies on neuropsychological tasks: the Stroop task evaluates inhibitory control (10), the Go/No-Go task assesses response inhibition (11), the N-back task measures working memory (12), and the Shape Trails Test (STT) evaluates cognitive flexibility (13).

The verbal fluency task (VFT) is a neuropsychological paradigm designed to assess speech fluency, which is divided into two subtypes: the semantic fluency test and the phonemic fluency test. The semantic fluency test is less cognitively demanding than the phonemic fluency test; the latter places greater reliance on EF, while the former requires the engagement of different semantic categories. The number of valid words generated during the VFT is a behavioral indicator of MCI (14).

Functional near-infrared spectroscopy (fNIRS) is a non-invasive neuroimaging technique with excellent temporal resolution that can capture changes in oxy-Hb and deoxyhemoglobin (deoxy-Hb)—changes that reflect cortical activation during task performance. The combination of fNIRS and VFT (fNIRS-VFT) is widely used to assess brain activation patterns in mental illnesses such as depression (15–18); however, few studies have examined the correlation between task-related prefrontal activation and cognitive function (19, 20).

Overall, the phonemic fluency test—a neuropsychological experimental paradigm— serves as a valid tool for evaluating EF, a cognitive process closely associated with the prefrontal lobe. In the present study, we employed fNIRS-VFT to examine prefrontal activation differences between the ED and non-ED groups, providing preliminary insights into the potential mechanisms underlying ED in ARCD.

## Materials and methods

### Subjects

All participants were recruited from our clinical trial entitled “Effects of cognitive-motor dual-task training on ARCD” (Registration No.: ChiCTR2200064684). Participants were included if they met all the following criteria: (1) aged 65 years or older; (2) MoCA score <26 (21); (3) evidence of gradual cognitive decline persisting for at least 6 months; (4) ability to perform Activities of Daily Living (ADLs) independently; (5) absence of previously diagnosed conditions known to cause cognitive decline. Participants were excluded if they met any of the following criteria: (1) A diagnosis of depression, anxiety, or other significant psychiatric disorders; (2) Dementia secondary to Parkinson’s disease, Alzheimer’s disease (AD), stroke, or other etiologies; (3) Failure to complete the fNIRS assessment.

We collected demographic information from all participants, including age, sex, ethnicity, and education level. Education levels were categorized as: Illiteracy, Primary School, Junior High School, Senior High School, Bachelor’s Degree, Master’s Degree, Doctor’s Degree. All participants were evaluated for global cognitive function using the MoCA. Memory function was assessed using the Auditory Verbal Learning Test Huashan Version (AVLT-H) (14) and the Digit Span Test (DST). Cognitive flexibility and cognitive processing speed were evaluated with the Shape Trails Test Part B (STT-B) and the Symbol Digit Modalities Test (SDMT) (15). The Modified Barthel Index (MBI) and the IADL scale were used to assess ADLs and IADL, respectively. All assessments were performed by a single professional rehabilitation physician. A total of 36 participants with ARCD were recruited for our study between October 2022 and October 2024 from nursing homes and communities in Beijing.

Higher scores on the MoCA, AVLT-H, DST, and SDMT indicate better cognitive function; a shorter completion time on the STT-B test indicates better EF in that domain. There are 5 points for visual-spatial/EF tasks in the MoCA scale, including trail making test, cube copying test, and clock drawing test, as well as 5 points for memory.

### Assignment to subgroups

Shape Trails Test Part B and the Montreal Cognitive Assessment Executive Function Subscale (MoCA-EF) were employed to evaluate EF. Participants were categorized into the ED group if their score on either of the two scales fell below the cutoff value; otherwise, they were categorized into the non-ED group. This approach was adopted to minimize the risk of missing older adults with relative EF decline. The cut-off value for STT-B was defined as a score indicating relative EF impairment (specifically, >1 SD below the age-corrected normative mean) (22). The MoCA includes five items assessing EF, for which no standardized cut-off score has been established; thus, we established the cut-off score by adopting the method described in previous studies (3, 23). First, we calculated the mean score on the EF subscales for all participants. Subsequently, the criterion for defining substantial relative EF impairment was set as a score at least 1.0 SD below this mean (3). This method yielded two subgroups: participants with relative EF impairment and those without. The study was approved by the Institutional Review Board of Beijing Rehabilitation Hospital, Capital Medical University. All participants provided written informed consent.

### fNIRS data acquisition

**Figure 1:**
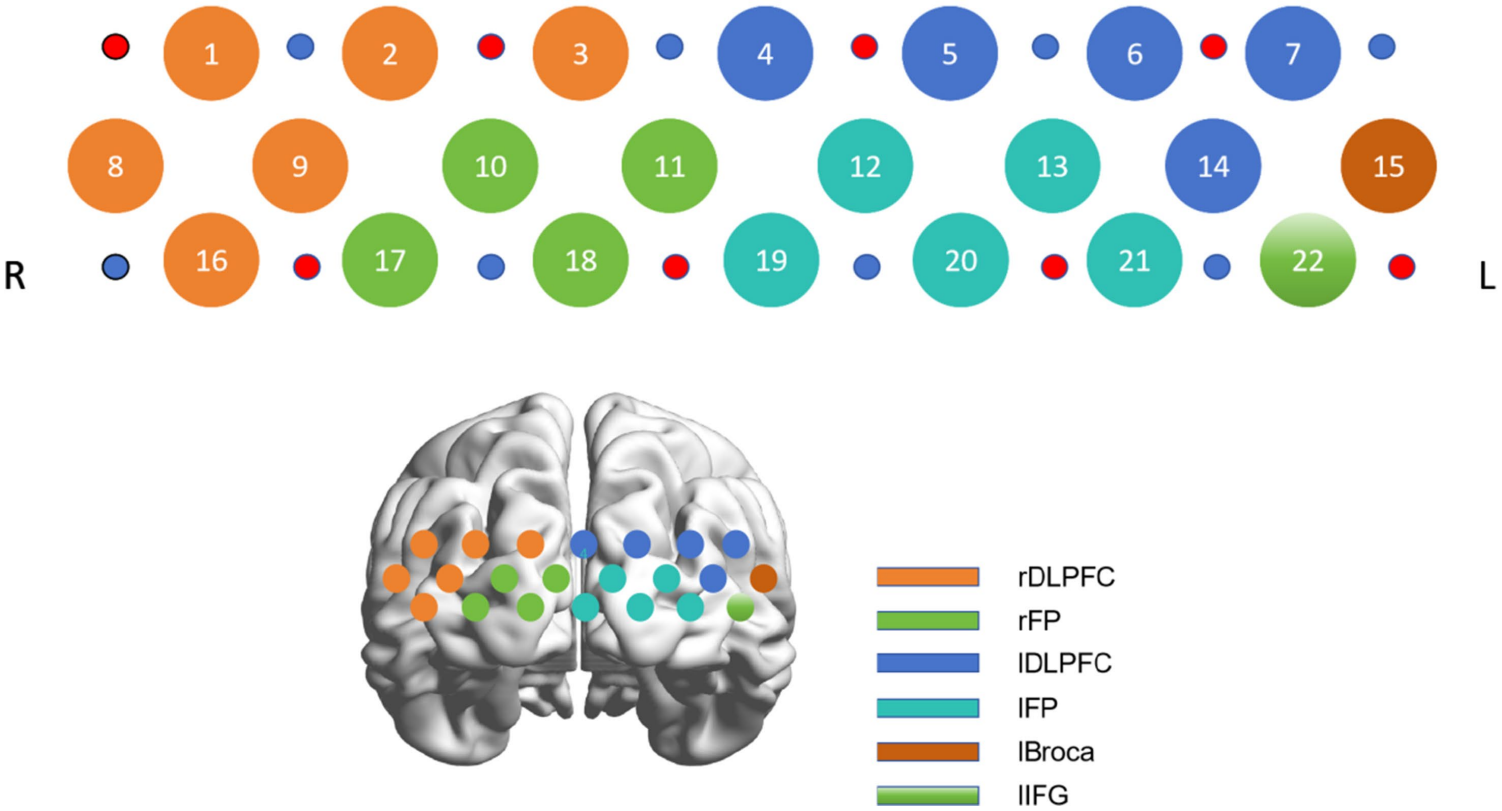
Probe distribution of frontal lobe (anterior view) and corresponding brain regions. Red denotes light sources, and blue denotes detector. DLPFC, dorsolateral prefrontal cortex, FP, frontopolar region, IFG, inferior frontal gyrus, L, left, R, right.

A 22-channel near-infrared optical imaging system with 8 sources and 8 detectors (ETG-4000, HITACHI, Japan), which covers the PFC, was utilized. The sampling frequency was set at 16.67 Hz, and the wavelengths were 730 nm and 850 nm. The light sources and detectors were designed according to an internationally used 10/20 electrode distribution system. The bottom probes were located along the PF1-PF2 line. The inter-probe distance was set to 3 cm. The distribution of channels and their corresponding brain regions is presented in Figure 1. This channel distribution has been employed in previous study (24).

The fNIRS recordings were performed concurrently with a VFT in a controlled environment with minimal acoustic noise and stable ambient lighting. The experimental paradigm comprised a 30 s pre-task resting baseline period, a 60 s verbal generation period, and a 30 s post-task recovery phase. Stimuli were presented on a display monitor positioned at a distance of 1 m away from the participants.

During the active task, participants were instructed to generate as many semantically meaningful phrases as possible, rapidly and accurately, using a series of specified Chinese characters [e.g., “白” (white), “大” (big), “天” (sky)]. Prior to the formal task, a practice trial using the character “门” (door) was administered to ensure task comprehension and standardization of the task procedure. The presentation order of the characters was fixed across all participants.

Each character was displayed for 2 s and subsequently followed by a central fixation cross (“+”) presented for 18 s. During both pre- and post-task rest periods, participants were instructed to engage in silent repetitive counting from 1 to 5 to minimize cognitive load and establish a consistent baseline (25).

### Data processing and analysis

NIRS_KIT (HuiChuang, Zhenjiang, China) (26) and BrainNet Viewer (27) were employed for data analysis and visualization. The raw light intensity data were first converted to optical density. Subsequently, physiological noise, such as signals arising from cardiac and respiratory activities, was eliminated using a bandpass filter (0.01–0.2 Hz). The filtered optical density signals were then transformed into concentrations of oxy-Hb and deoxy-Hb based on the modified Beer-Lambert law. For all subsequent analyses, oxy-Hb was selected over deoxy-Hb due to its superior signal-to-noise ratio, as documented in previous research (18). A baseline was derived from the 30 s period preceding the task, which was used to calculate the mean changes in oxy-Hb concentration across all 22 prefrontal channels during the VFT.

The clinical data were analyzed using IBM SPSS Statistics version 26.0. Data normality was tested via the Shapiro–Wilk test. Continuous variables including age and scores on cognitive and ADL-related scales (MoCA, AVLT-H, DST, SDMT, STT-B, MBI and IADL) were analyzed using an unpaired *t*-test. Categorical variables such as sex, education, and clinical variables (hypertension, diabetes, alcohol consumption, smoking status, sedentary lifestyle) were analyzed by Fisher’s exact test. An unpaired *t*-test was used to compare the difference in oxy-Hb changes within the PFC during the VFT task between the ED and non-ED groups of ARCD participants. Paired *t*-test was used to compare oxy-Hb changes between non-task and VFT phases to identify task-related brain region activation. Spearman correlation analysis was performed between oxy-Hb changes and the MoCA/MoCA-EF/SDMT/STT-B scores in all the participants. If the data did not follow a normal distribution, the Mann–Whitney *U* test was used instead. *p*-values of all the within- and between-group comparisons were corrected by the false discovery rate (FDR) method. We also calculated the effect size (ES) for the group differences of oxy-Hb changes in each channel in order to evaluate whether the differences are independent of sample size using Cliff’s Delta (*δ*) – a non-parametric ES measure designed to quantify the magnitude of difference between two independent groups, particularly for data that violates the normality assumption. According to the ES classification criteria proposed by Romano et al. (28), an ES of <0.147 is classified as negligible; an ES ranging from 0.147 to 0.33 is defined as a small effect size; an ES between 0.33 and 0.474 is categorized as a medium effect size; and an ES of >0.474 is regarded as a large effect size. A *p*-value less than 0.05 was considered statistically significant, and all *p*-values were two-tailed.

## Results

### Demographic and clinical characteristics

No statistically significant differences were observed between the ED and non-ED groups in terms of sex, education level, AVLT-H scores, DST scores, IADL scores, frequency of smoking and drinking, prevalence of hypertension, or sedentary behavior. All participants achieved an MBI score of 100. Compared with the non-ED group, the ED group had a significantly older age (*p* < 0.05) and significantly lower MoCA scores (*p* < 0.05). Additionally, the prevalence of type 2 diabetes mellitus was significantly higher in the ED group than in the non-ED group (*p* < 0.05). Detailed data are presented in Table 1.

**Table 1:** Demographic characteristics of participants in each group.

Variable	ED (*n* = 18)	Non-ED (*n* = 18)	*t*/*z*	*p*
Age (*M* ± SD)	78.44 ± 7.52	73.33 ± 5.58	2.316	0.027^*^
Sex (male/female)	7/11	9/9		0.738
MoCA [*M* (P25, P75)]	22 (20, 23)	24 (21, 25)	−2.396	0.017^*^
Education				
Illiteracy	0	0		0.742
Primary school	0	2		
Junior high school	4	3		
Senior high school	3	3		
Bachelor’s degree	11	10		
Master’s degree	0	0		
AVLT-H				
AVLT-D (*M* ± SD)	4.3 ± 2.7	5.1 ± 2.2	−0.913	0.368
AVLT-R [*M* (P25, P75)]	21.0 (19, 22.5)	21.5 (20.0, 22.8)	−0.272	0.785
DST				
Forward [*M* (P25, P75)]	7 (6, 8)	7 (6, 8)	−0.267	0.789
Backward [*M* (P25, P75)]	4 (3, 5)	5 (3, 5)	−0.588	0.556
SDMT (*M* ± SD)	20.9 ± 10.8	33.8 ± 8.7	−3.860	0.000^***^
STT-B (*M* ± SD)	292.5 ± 80.3	166.3 ± 50.0	5.615	0.000^***^
Smoking status				
Yes	1	0		0.658
No	14	16		
Quit	3	2		
Alcohol consumption				
Yes	3	3		1.000
No	14	14		
Quit	1	1		
Hypertension				
Yes	12	7		0.091
No	6	11		
Type 2 diabetes				
Yes	8	2		0.03^*^
No	10	16		
Sedentary lifestyle				
>5 h/day	3	5		0.345
≤5 h/day	15	13		
IADL, *M* (P25, P75)	22 (21, 24)	23 (23, 24)	0.833	0.491

ED, executive dysfunction; non-ED, non-executive dysfunction; MoCA, Montreal cognitive assessment; AVLT-H, Auditory Verbal Learning Test Huashan Version; DST, Digit Span Test; D, delayed free recall; R, recognition; SDMT, Symbol Digit Modalities Test; STT-B, Shape Trails Test B; *M*, mean or median; SD, Standard deviation; P25, 25th percentile; P75, 75th percentile; IADL, Instrumental Activities of Daily Living. ^*^Indicates *p* < 0.05; ^***^Indicates *p* < 0.001.

### Behavioral results: verbal fluency task outcomes

**Figure 2:**
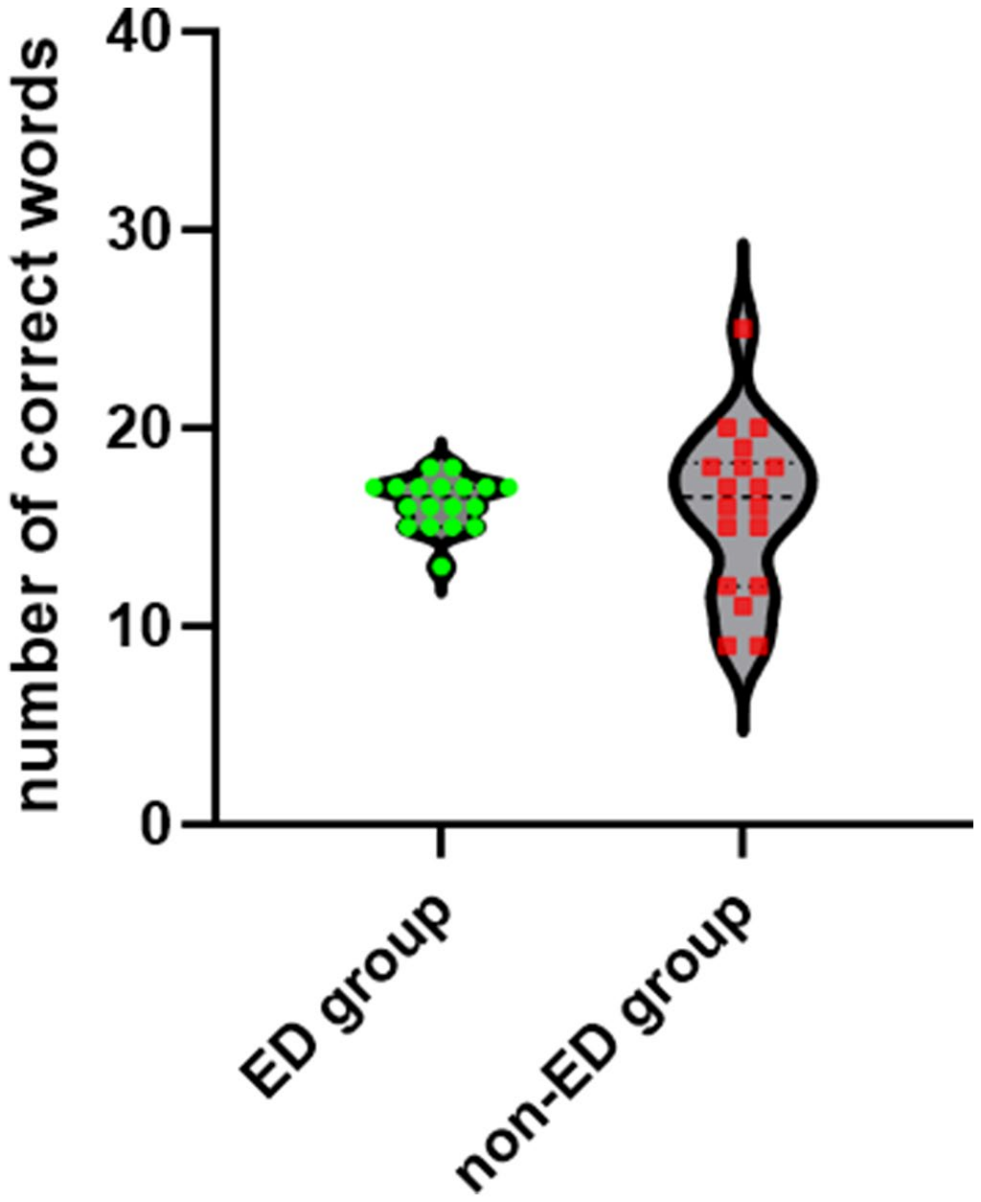
The number of correct words during a verbal fluency task. There was no significant difference between the ED group and the non-ED group.

The number of correct words generated by the two participant groups during the VFT was analyzed using the Mann–Whitney *U* test. Results revealed no significant difference between the two groups in the total number of correct words (*z* = −0.192, *p* = 0.847), as shown in Figure 2.

### Brain activation in the ED and non-ED group

#### Oxy-Hb concentration changes in the non-ED group

**Figure 3:**
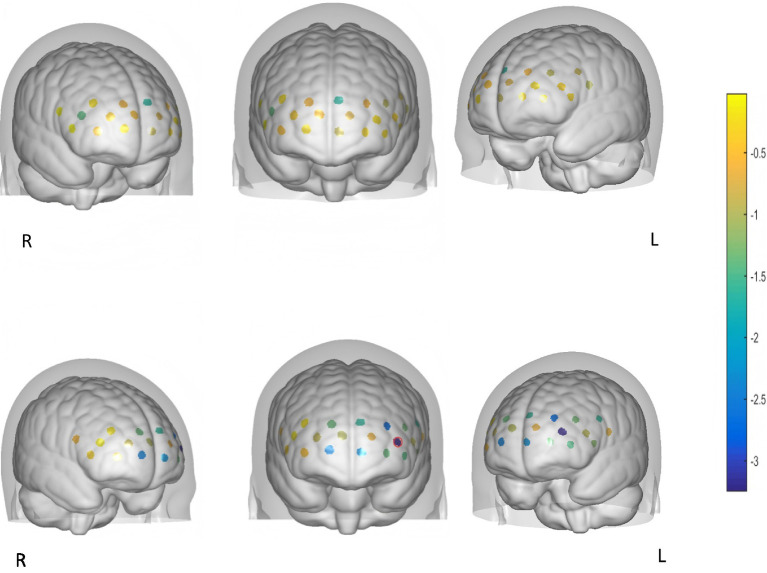
Brain activation of the ED group and non-ED group: *z*-value maps with FDR-corrected *p*-values <0.05 circled in red.

**Figure 4:**
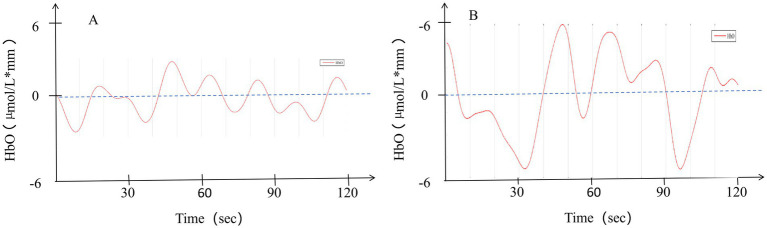
Time course of relative oxy-Hb changes in a non-ED group **(A)** and an ED group **(B)**. HbO, Oxyhemoglobin.

Non-ED group: Since not all oxy-Hb concentration changes followed a normal distribution, a non-parametric test for two related samples was employed. Statistically significant differences were observed in Channel 5 (Ch5), Channel 13 (Ch13), Channel 18 (Ch18), and Channel 19 (Ch19), which are located in the left dorsolateral prefrontal cortex and bilateral frontal pole (FP). After FDR correction, a statistically significant difference persisted only in Ch13, which is located in the lFP. No statistically significant differences were detected in the remaining channels, as shown in Table 2 and Figure 3. Additionally, to illustrate the typical response pattern, the oxy-Hb time-response curve derived from one channel of a single participant is shown in Figure 4A. It appeared that the oxy-Hb concentration changes exhibited mild magnitude of variation, pronounced fluctuations, along with the absence of distinct peaks and the lack of an initial dip throughout the entire duration of the task paradigm.

**Table 2:** Brain activation of the non-ED group at each channel (*n* = 18).

Oxy-Hb	*M* (P25, P75) (μmol/L * mm)	*z*	*p*	*p* (FDR)
Baseline 1	−0.2 (−1.5, 1.1)	−0.370	0.711	0.823
Task 1	−0.1 (−0.8, 0.5)			
Baseline 2	0.1 (−0.6, 0.6)	−0.196	0.845	0.885
Task 2	0.2 (−0.4, 0.5)			
Baseline 3	−0.3 (−0.6, 0.3)	−1.372	0.17	0.468
Task 3	0.1 (−0.1, 0.3)			
Baseline 4	−0.3 (−1.1, 0.2)	−1.633	0.102	0.449
Task 4	0.2 (0, 0.5)			
Baseline 5	−0.3 (−1.0, 0)	−2.722	0.006^**^	0.066
Task 5	0.2 (−0.1, 0.5)			
Baseline 6	−0.2 (−0.9, 0.1)	−1.328	0.184	0.405
Task 6	0.1 (−0.2, 0.3)			
Baseline 7	−0.2 (−1.5, 0.4)	−1.590	0.112	0.411
Task 7	0.1 (−0.3, 0.5)			
Baseline 8	0.2 (−1.1, 1.5)	−0.544	0.586	0.758
Task 8	−0.2 (−1.4, 0.6)			
Baseline 9	0 (−1.3, 1.3)	−0.152	0.879	0.879
Task 9	−0.3 (−0.7, 0.6)			
Baseline 10	0 (−0.4, 0.3)	−0.893	0.372	0.585
Task 10	0.1 (−0.2, 0.4)			
Baseline 11	−0.3 (−0.7, 0.4)	−1.024	0.306	0.561
Task 11	0.2 (−0.3, 0.4)			
Baseline 12	−0.1 (−0.7, 0.3)	−0.632	0.528	0.726
Task 12	0 (0, 0.1)			
Baseline 13	−0.4 (−0.9, -0.1)	−3.245	0.001^**^	0.022^*^
Task 13	0.4 (0.1, 10)			
Baseline 14	0 (−0.6, 0.8)	−0.936	0.349	0.591
Task 14	0.4 (−0.2, 0.6)			
Baseline 15	0.1 (−1.5, 0.8)	−0.719	0.472	0.692
Task 15	0.2 (−0.1, 0.7)			
Baseline 16	0 (−1.7, 0.9)	−0.327	0.744	0.818
Task 16	0 (−0.9, 0.5)			
Baseline 17	0 (−0.5, 0.4)	−0.501	0.616	0.753
Task 17	0 (−0.4, 0.3)			
Baseline 18	0 (−1.1, 0.4)	−2.461	0.014^*^	0.103
Task 18	0.3 (0.2, 0.6)			
Baseline 19	−0.2 (−0.7, 0)	−2.330	0.02^*^	0.11
Task 19	0.2 (0, 0.4)			
Baseline 20	−0.1 (−1.0, 0.1)	−1.372	0.17	0.416
Task 20	0.3 (−0.1, 0.6)			
Baseline 21	0 (−1.7, 0.4)	−1.459	0.145	0.456
Task 21	0.3 (−0.3, 0.7)			
Baseline 22	−0.1 (−1.1, 0.6)	−1.198	0.231	0.462
Task 22	0.2 (−0.2, 0.7)			

^*^Indicates *p* < 0.05; ^**^Indicates *p* < 0.01; oxy-Hb, oxyhemoglobin; FDR, false discovery rate; Baseline, Oxy-Hb concentration changes during silent counting from 1 to 5; Task, Oxy-Hb concentration changes during the VFT; M, median; P25, 25th percentile; P75, 75th percentile; μmol/L * mm, micromole per liter per millimeter.

#### Oxy-Hb concentration changes of the ED group

The relative concentration changes of oxy-Hb at baseline and during the VFT task across 22 channels did not all show a normal distribution. A statistically significant difference was observed in Ch1, but no statistically significant differences remained after FDR correction. For the other channels, no statistically significant differences were found, as presented in Table 3 and Figure 3. Additionally, the oxy-Hb time-response curve from the ED group is presented in Figure 4B. In comparison to the curve in Figure 4A, this curve also exhibited mild variations in magnitude, pronounced fluctuations, and a complete absence of notable amplitude.

**Table 3:** Brain activation of the ED group at each channel (*n* = 18).

Oxy-Hb	*M* (P25, P75) (μmol/L * mm)	*z*	*p*	*p* (FDR)
Baseline 1	0.3 (−0.1, 0.6)	−2.504	0.012^*^	0.264
Task 1	−0.1 (−0.5, 0.1)			
Baseline 2	0.1 (−0.4, 0.6)	−0.240	0.811	1
Task 2	0.1 (−0.2, 0.3)			
Baseline 3	−0.1 (−0.9, 0.4)	−0.588	0.557	1
Task 3	0 (−0.5, 0.4)			
Baseline 4	−0.4 (−1.1, 0.2)	−1.677	0.094	1
Task 4	0.2 (−0.2, 0.5)			
Baseline 5	−0.3 (−1, 0.5)	−0.588	0.557	1
Task 5	0 (−0.3, 0.2)			
Baseline 6	0 (−0.5, 0.4)	−0.936	0.349	1
Task 6	0.2 (−0.2, 0.5)			
Baseline 7	0.1 (−0.9, 0.5)	−0.849	0.396	1
Task 7	0 (−0.2, 0.7)			
Baseline 8	0.1 (−0.7, 0.8)	−0.065	0.948	0.993
Task 8	0 (−0.4, 0.7)			
Baseline 9	0.2 (−0.3, 0.7)	−1.415	0.157	1
Task 9	−0.2 (−0.7, 0.3)			
Baseline 10	0 (−0.5, 0.5)	−0.414	0.679	1
Task 10	0 (−0.3, 0.5)			
Baseline 11	0 (−0.3, 0.1)	−0.501	0.616	1
Task 11	0 (−0.3, 0.2)			
Baseline 12	−0.1 (−0.8, 0.5)	−0.762	0.446	1
Task 12	0.1 (−0.1, 0.2)			
Baseline 13	0.1 (−0.5, 0.2)	−0.240	0.811	0.991
Task 13	0.1 (−0.5, 0.4)			
Baseline 14	0.2 (−0.5, 0.6)	−0.327	0.744	1
Task 14	−0.1 (−0.5, 0.4)			
Baseline 15	−0.4 (−0.9, 0.3)	−0.980	0.327	1
Task 15	0.1 (−0.4, 0.5)			
Baseline 16	0 (−0.8, 0.4)	−0.022	0.983	0.983
Task 16	−0.1 (−0.3, 0.6)			
Baseline 17	0.1 (−0.5, 0.4)	−0.501	0.616	1
Task 17	−0.1 (−0.3, 0.2)			
Baseline 18	0.3 (−0.6, 0.6)	−0.240	0.811	0.939
Task 18	0.1 (−0.4, 0.4)			
Baseline 19	−0.1 (−1.0, 0.1)	−0.893	0.372	1
Task 19	0 (−0.3, 0.7)			
Baseline 20	0.2 (−0.9, 0.6)	−0.283	0.777	1
Task 20	0 (−0.4, 0.6)			
Baseline 21	−0.2 (−0.6, 0.3)	−0.240	0.811	0.892
Task 21	0 (−0.6, 0.3)			
Baseline 22	0 (−0.5, 0.4)	−0.283	0.777	1
Task 22	−0.1 (−0.8, 0.6)			

^*^Indicates *p* < 0.05; oxy-Hb, oxyhemoglobin; FDR, false discovery rate; Baseline, Oxy-Hb concentration changes during silent counting from 1 to 5; Task, Oxy-Hb concentration changes during the VFT; M, median; P25, 25th percentile; P75, 75th percentile; μmol/L * mm, micromole per liter per millimeter.

#### The group comparison for relative changes of brain activation between the ED group and non-ED group

Not all relative concentration changes of oxy-Hb across the 22 channels followed a normal distribution. Therefore, we employed an independent-samples Mann–Whitney *U* test for subsequent analyses, which revealed a statistically significant difference in oxy-Hb concentration changes for Ch13 (*z* = −2.088, *p* = 0.035). However, this significance was lost following FDR correction. Additionally, no statistically significant differences in oxy-Hb concentration changes were observed in the remaining channels (all *p* > 0.05). Detailed results for all 22 channels are provided in Table 4.

**Table 4:** Comparison of oxy-Hb concentration changes between the two groups.

Ch	Group	*M* (P25, P75) (μmol/L * mm)	*z*	*p*	*p* (FDR)	*δ*
1	ED	−0.4 (−0.8, −0.4)	−0.791	0.429	1	−0.15
	Non-ED	0.2 (−2.0, 0.2)				
2	ED	0 (−0.6, 0)	−0.411	0.681	1	−0.08
	Non-ED	0.4 (−1.1, 0.4)				
3	ED	0 (−0.0006, 0)	−0.348	0.728	0.942	−0.07
	Non-ED	0.4 (−0.3, 0.4)				
4	EF	0.6 (−0.3, 0.6)	−0.127	0.899	0.989	−0.02
	Non-ED	0.7 (−0.3, 0.7)				
5	ED	0.3 (−0.8, 0.3)	−0.981	0.327	1	−0.19
	Non-ED	0.5 (0.1, 0.5)				
6	ED	0.2 (−0.4, 0.2)	−0.538	0.591	1	−0.1
	Non-ED	0.3 (−0.3, 0.3)				
7	ED	−0.1 (−0.7, −0.1)	−0.696	0.486	1	−0.14
	Non-ED	0.2 (−0.6, 0.2)				
8	ED	−0.2 (−1.1, −0.2)	−0.38	0.704	0.968	−0.07
	Non-ED	−0.1 (−2.4, −0.1)				
9	ED	−0.3 (−0.9, −0.3)	−0.411	0.681	1	−0.08
	Non-ED	0 (−2.0, 0)				
10	ED	0 (−0.6, 0)	−0.285	0.776	0.948	−0.06
	Non-ED	0.2 (−0.4, 0.2)				
11	ED	0 (−0.1, 0)	−1.297	0.195	1	−0.25
	Non-ED	0.6 (−0.6, 0.6)				
12	ED	0.1 (−0.5, 0.1)	−0.127	0.899	0.942	−0.02
	Non-ED	0.1 (−0.3, 0.1)				
13	ED	0.2 (−0.8, 0.2)	−2.088	0.037^*^	0.814	−0.41
	Non-EF	0.8 (0.1, 0.8)				
14	ED	−0.2 (−1.3, −0.2)	−0.918	0.359	1	−0.18
	Non-ED	0.3 (−0.6, 0.3)				
15	ED	0.4 (−0.7, 0.4)	−0.158	0.874	1	−0.03
	Non-ED	0.3 (−0.7, 0.3)				
16	ED	−0.2 (−0.9, −0.2)	−0.063	0.95	0.95	−0.01
	Non-ED	0.1 (−1.4, 0.1)				
17	ED	−0.1 (−0.8, −0.1)	−0.601	0.548	1	−0.12
	Non-ED	0.2 (−0.5, 0.2)				
18	ED	−0.5 (−0.8, −0.5)	−1.614	0.107	1	−0.31
	Non-ED	0.4 (0, 0.4)				
19	ED	0.1 (−0.6, 0.1)	−0.601	0.548	1	−0.12
	Non-ED	0.4 (0, 0.4)				
20	ED	−0.3 (−1.0, −0.3)	−0.854	0.393	1	−0.17
	Non-ED	0.5 (−0.4, 0.5)				
21	ED	0 (−0.9, 0)	−0.949	0.343	1	−0.19
	Non-ED	0.3 (−0.5, 0.3)				
22	ED	−0.1 (−1.1, 1.6)	−0.411	0.696	1	−0.08
	Non-ED	0.3 (−0.7, 1.4)				

^*^Indicates *p <* 0.05; Ch, channel; ED, executive dysfunction; Non-ED, non-executive dysfunction; FDR, false discovery rate; M, median; P25, 25th percentile; P75, 75th percentile.

### Correlational analysis between cognitive function and oxy-Hb concentration changes

**Figure 5:**
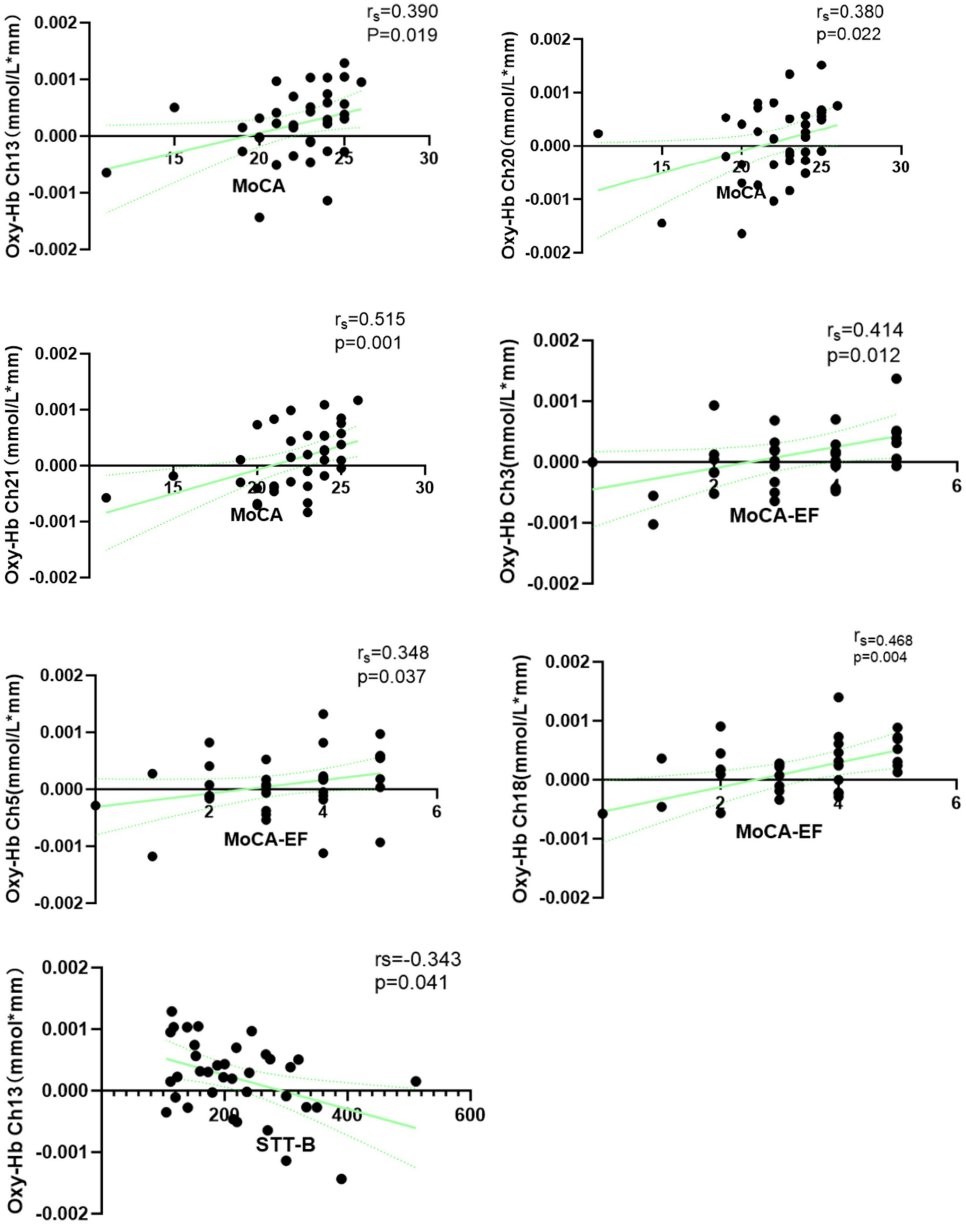
Spearman correlation between oxy-Hb concentration changes and cognition in age-related cognitive decline.

Spearman’s rank correlation analysis was performed between relative oxy-Hb concentration changes in various channels and scores on the MoCA, MoCA-EF, SDMT, and STT-B. Statistically significant positive correlations were observed between MoCA scores and average oxy-Hb concentration changes in Ch13 (*r_s_* = 0.390, *p* = 0.019), Ch20 (*r_s_* = 0.380, *p* = 0.022), and Ch21 (*r_s_* = 0.515, *p* = 0.001) – all of which are located in the lFP. After FDR correction, only the correlation between MoCA scores and average oxy-Hb concentration changes in Ch21 remained statistically significant (*r_s_* = 0.515, *p* = 0.022). Additionally, statistically significant positive correlations were found between MoCA-EF scores and average oxy-Hb concentration changes in Ch3 (*r_s_* = 0.414, *p* = 0.012), Ch5 (*r_s_* = 0.348, *p* = 0.037), and Ch18 (*r_s_* = 0.468, *p* = 0.004). Ch3 is located in the rDLPFC, Ch5 in the lDLPFC, and Ch18 in the lFP; however, none of these correlations remained statistical significance after FDR correction. For the STT-B, a statistically significant negative correlation was observed between its scores and average oxy-Hb concentration changes in Ch13 (*r_s_* = −0.343, *p* = 0.041; located in the lFP), but this correlation no longer reached statistical significance following FDR correction. These results are shown in Figure 5. No statistically significant correlations were detected between average oxy-Hb concentration changes in all channels and either SDMT scores or age.

## Discussion

The ARCD is associated with advancing age. In addition to age, it may also be related to other aging-related comorbidities, such as hypertension and diabetes. In this study, individuals with ARCD were categorized into the ED group and non-ED group based on performance on the MoCA EF subscale and STT-B. Specifically, the STT-B can be used to evaluate cognitive flexibility, while the clock drawing test within the MoCA can be used to assess planning and working memory. Participants were considered to have ED if their score on either of these two measures fell below the predefined cutoff. No statistically significant differences were observed between the two groups in terms of sex, education level, prevalence of hypertension, frequency of smoking and alcohol intake, or sedentary behavior; nor were there significant differences in memory assessment scores, specifically those from the DST and AVLT-H. However, the ED group was significantly older than the non-ED group, indicating that age may be a risk factor for impaired EF in individuals with ARCD. These findings are consistent with a previous study indicating that EF performance decreases after the age of 65 years (29). This also highlights that EF in ARCD warrants further investigation. We also observed that the incidence of type 2 diabetes in the ED group was higher than that in the non-ED group, suggesting that type 2 diabetes may also be a risk factor for ARCD. Previous studies have demonstrated that diabetes can induce cognitive decline, including impairments in attention, EF, and processing speed (30). Although scores on EF measures, total MoCA scores, and age differed between the two groups, our analyses revealed no significant between-group difference in the total number of words generated during the VFT. This unexpected null finding is most plausibly explained by the multifactorial nature of the VFT performance. Word generation in this task is influenced not only by core cognitive capacities, but also by contextual and individual non-cognitive factors, such as cultural background, emotional state, and task motivation. This finding suggests that the VFT lacked sufficient sensitivity to distinguish between ED and non-ED individuals with ARCD, despite the fact that the VFT has been shown to be a sensitive indicator for distinguishing normal cognition from MCI (14).

Intra-group comparison revealed that no PFC channels were activated in the ED group, whereas only one channel located at the left frontal pole was activated in the non-ED group of ARCD participants. Notably, previous studies have demonstrated that the phonemic fluency test is associated with the PFC, especially the left PFC (31), and it not only activates multiple channels of the PFC in normal elderly individuals and elderly individuals with Parkinson’s disease without cognitive impairment (14, 32), but also activates small patches of the right orbitofrontal cortex in some healthy female adults (31). Consistent with these established findings, our study found that the left PFC was activated in the non ED group. However, our results further highlighted a marked reduction in frontal lobe activation among older adults with ARCD: in stark contrast to the single-channel activation observed in the non-ED group, no prefrontal channels exhibited significant activation in the ED group at all. Beyond cortical activation profiles, our analysis of oxy-Hb concentration curves during the VFT also identified distinct response features, characterized by multiple fluctuations and negligible amplitude variation—a pattern that diverges from the response dynamics reported in prior research (16). Plausibly, this atypical hemodynamic response may be linked to two interrelated factors: aging itself, which is known to induce reduced cerebral hemodynamic responses (33), and the cognitive decline inherent to ARCD (19). Collectively, these observations align with prior evidence indicating that patients with MCI also exhibit diminished PFC activity during cognitive tasks (9, 19, 34).

Inter-group comparisons revealed that the relative change in oxy-Hb concentration at Ch13, located in the left frontal pole, was marginally higher in the non-ED group than the ED group, yet, this between-group discrepancy failed to attain statistical significance following FDR correction. Collectively, these findings tentatively imply that cortical activity within the left frontal hemisphere is attenuated in ED older adults compared with their non-ED counterparts, albeit such a trend did not reach statistical significance. This null result might plausibly stem from a confluence of interrelated factors: specifically, the blunted cerebral hemodynamic responsiveness characteristic of older adults with cognitive decline (19), may have induced a ceiling effect, narrowing the gap in prefrontal activation levels between the two subgroups and thereby obscuring statistically detectable differences. Furthermore, as a preliminary exploratory investigation, the modest sample size of our ARCD cohort likely limits statistical power to detect subtle yet potentially biologically meaningful between-group variations in prefrontal hemodynamic responses. In the future, these results may offer valuable insights to inform subsequent investigations, and the robustness of this trend awaits further verification in cohorts with larger sample sizes.

Previous studies have shown that NIRS-derived regional cerebral activation exhibits a positive correlation with the number of words generated in the VFT (25). Our study found that although there were certain differences in NIRS-derived regional cerebral activation between the two groups, the VFT performance of the two groups was comparable. This may suggest that the observed differences in NIRS activation may be attributed to cognitive impairment itself, and this effect is independent of the VFT performance. Similar findings have been reported in other studies—for instance, those focusing on depression (35, 36)—where no differences were observed in VFT performance, yet statistically significant differences were noted in NIRS-derived activation.

Our study demonstrated that MoCA scores exhibited a positive correlation with oxy-Hb concentration changes across most channels located at lFP. Specifically, EF scores, which are a subcomponent of the MoCA, showed a positive correlation with oxy-Hb changes in a small portion of the lFP cortex and the bilateral DLPFC, while STT-B scores were negatively correlated with those in a small portion of the lFP. However, after FDR correction, only the correlation between MoCA scores and oxy-Hb changes of a small portion of lFP remained significantly significant, indicating that the fNIRS-VFT might be used as an auxiliary tool for cognition assessment. Similar to previous studies, prefrontal hemodynamic response by fNIRS was related to cognitive decline (32, 34, 37), and this technique has also been used to distinguish between individuals with MCI and dementia (34). As an exploratory study, the present work highlights that the correlation between EF and prefrontal cortical activity warrants further investigation.

There are several limitations to our study. Firstly, the ED group and non-ED group were grouped by STT-B and MoCA-EF. However, inhibitory control—one dimension of EF—wasn’t assessed. Consequently, there may have been some elderly who actually had ED but were misclassified into the non-ED group, which could have contributed to null results. Secondly, there were significant differences in age and diabetes incidence between the two groups. According to previous studies, diabetes exerts little impact on oxy-Hb changes in the prefrontal lobe (38); however, age may influence prefrontal oxy-Hb changes during the VFT (39). Thus, it remains difficult to rule out the potential confounding effect of age. To address this limitation, elderly individuals with no cognitive decline should be enrolled as a control group in future studies. Thirdly, while ARCD was the target population of this study, relying solely on MoCA scores and medical history posed challenges for excluding cognitive decline with identified etiologies. This limitation arises because such etiologically defined cognitive changes may overlap with ARCD in clinical manifestations, yet cannot be differentiated by MoCA or basic medical history alone. Finally, we did not perform an *a priori* sample size calculation, Nevertheless, the concentration change of oxy-Hb at Ch13 was higher in the non-ED group than in the ED group, suggesting our sample did capture the effect in this instance; however, we cannot rule out that a larger sample might yield more stable, generalizable results.

In conclusion, the present study demonstrated that older adults with ARCD exhibited attenuated prefrontal cortical activity during the VFT. Notably, no statistically significant between-group difference in prefrontal activation was detected between the ED and non-ED subgroups of ARCD participants, indicating that EF-related subtyping may not correspond to overt alterations in prefrontal hemodynamic responses during VFT in this ARCD cohort. Furthermore, MoCA scores were positively associated with changes in oxy-Hb concentration in the left frontal pole, suggesting that fNIRS-derived PFC activation may serve as a potential biomarker for global cognitive decline in ARCD. However, if quantitative analysis of fNIRS-VFT data is to be applied in clinical cognitive assessment, a longitudinal study would be required to validate its utility and stability.

## Data Availability

The raw data supporting the conclusions of this article will be made available by the authors, without undue reservation.
